# Theranostic Biomarkers for Schizophrenia

**DOI:** 10.3390/ijms18040733

**Published:** 2017-03-30

**Authors:** Matea Nikolac Perkovic, Gordana Nedic Erjavec, Dubravka Svob Strac, Suzana Uzun, Oliver Kozumplik, Nela Pivac

**Affiliations:** 1 Rudjer Boskovic Institute, Division of Molecular Medicine, 10000 Zagreb, Croatia; mnikolac@irb.hr (M.N.P.); gnedic@irb.hr (G.N.E.); dsvob@irb.hr (D.S.S.); 2 Clinic for Psychiatry Vrapce, 10090 Zagreb, Croatia; suzana.uzun@gmail.com (S.U.); okozumplik@hotmail.com (O.K.)

**Keywords:** schizophrenia, biomarkers, diagnostic, prognostic and theranostic biomarkers, inflammatory, stress-related, metabolic, neurotrophic and neurotransmitter biomarkers

## Abstract

Schizophrenia is a highly heritable, chronic, severe, disabling neurodevelopmental brain disorder with a heterogeneous genetic and neurobiological background, which is still poorly understood. To allow better diagnostic procedures and therapeutic strategies in schizophrenia patients, use of easy accessible biomarkers is suggested. The most frequently used biomarkers in schizophrenia are those associated with the neuroimmune and neuroendocrine system, metabolism, different neurotransmitter systems and neurotrophic factors. However, there are still no validated and reliable biomarkers in clinical use for schizophrenia. This review will address potential biomarkers in schizophrenia. It will discuss biomarkers in schizophrenia and propose the use of specific blood-based panels that will include a set of markers associated with immune processes, metabolic disorders, and neuroendocrine/neurotrophin/neurotransmitter alterations. The combination of different markers, or complex multi-marker panels, might help in the discrimination of patients with different underlying pathologies and in the better classification of the more homogenous groups. Therefore, the development of the diagnostic, prognostic and theranostic biomarkers is an urgent and an unmet need in psychiatry, with the aim of improving diagnosis, therapy monitoring, prediction of treatment outcome and focus on the personal medicine approach in order to improve the quality of life in patients with schizophrenia and decrease health costs worldwide.

## 1. Vulnerability and Resilience in Mental Disorders

There are numerous environmental and biological factors that interact with genetic factors and contribute to vulnerability or resilience with respect to the development of mental disorders [1]. Most psychiatric disorders, including schizophrenia, develop in vulnerable subjects who failed to cope with stress and do not achieve positive outcomes when faced with adversities and stressors [2]. Resilience is modulated by diverse systems (neurotransmitters, neurotrophic factors, hypothalamic–pituitary–adrenal (HPA) axis, immune system, autonomic nervous system, oxidative stress and metabolic markers), working together to recover from various stressors and stressful experiences and to achieve a positive adaptation after exposure to stressors that are presenting as threats to homeostasis [3,4]. Resilience may help understanding the heterogeneous therapeutic outcomes in schizophrenia [4]. One of the major systems involved in the regulation of the stress is the HPA axis, which shows enhanced activity (i.e., increased release of corticotrophin releasing factor (CRH), adrenocorticotrophic hormone (ACTH) and cortisol) as a response to stress [5,6]. The HPA axis is self-regulated, with a negative feedback mechanism which inhibits HPA axis activity; and after the system responses to stress or challenge, the HPA axis returns to its “normal” activity, i.e., homeostasis [5,6].

## 2. Schizophrenia

Schizophrenia is highly heritable, chronic, severe, disabling neurodevelopmental brain disease with heterogeneous genetic and neurobiological background. Symptoms of schizophrenia include positive, negative, cognitive and mood symptoms. Positive symptoms consist of psychotic symptoms, such as hallucinations, delusions, and disorganized speech and behavior. Negative symptoms include reduced emotions, poverty of speech, and loss of interests and drive, while cognitive symptoms comprise of deficits in working memory, attention and in executive functions, such as the ability to organize and abstract. Mood symptoms consist of depressive, cheerful or sad moods. Schizophrenia is a complex, multifactorial and polygenic mental disorder, estimated to affect 1% of population worldwide [1,2,7]. Stress, which modifies the HPA function, has been strongly implicated in the etiology of schizophrenia [8]. The neural-diathesis stress model of schizophrenia hypothesizes that schizophrenia develops as a consequence of the HPA axis dysregulation, and that different neurotransmitters and neuroimmune factors, neuroanatomical structures, pathways and connectivity, and genetic, epigenetic, neurodevelopmental and environmental factors modulate the main stress system (i.e., HPA axis activity) in vulnerable subjects exposed to stress [9,10,11].

The development of schizophrenia is mediated by numerous risk factors [2]: prenatal and perinatal complications, paternal older age, male gender, living in the urban environment, migration status, drug abuse (especially cannabis, amphetamine, methamphetamine and cocaine), and exposure to stressors (physical abuse, sexual abuse, maltreatment and bullying). The etiology of schizophrenia is far from clear, but family, twin and adoption studies all point to the major role of genetic factors, among other contributing factors, in its etiology [1]. Genetic factors might be associated with vulnerability to the disorder (i.e., they represent risk factors) in the presence of negative environmental influences (gene x environment interaction), and are proposed to be called plasticity variants, since they moderate susceptibility to both negative as well as positive environmental influences [1]. However, like in other complex mental disorders, it is assumed that development of schizophrenia is associated with complex interactions between various genetic risk factors, environmental factors, and exposure to early traumatic experiences, which all together modulate/influence the long-lasting neurodevelopmental changes in the neural circuits and neural pathways of the central nervous system (CNS) and alter homeostasis. When these factors overcome resilient defense mechanisms, schizophrenia will develop in vulnerable subjects. Therefore, genetic predisposition in accord with negative environmental stimuli will trigger development of schizophrenia; while on the other hand, without adverse environmental stimuli, genetic predisposition alone will not be responsible for development of the disease [1].

## 3. Biomarkers

**Figure 1:**
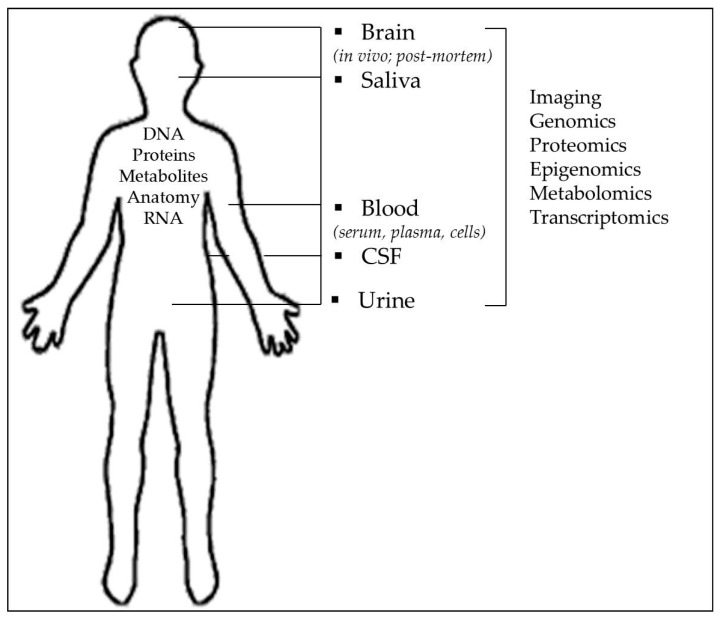
Biomarkers that can be measured or determined from the post-mortem brain or using in vivo neuroimaging studies, as well as from peripheral cells and easily available body fluids such as plasma/serum, urine or cerebrospinal fluid (CSF).

Due to diverse clinical phenotypes, symptoms and presentation, and heterogeneity of the overlapping symptoms and functional deficits in schizophrenia [2,12], its underlying biological background is poorly understood. Therefore, studies are focused on investigations of smaller, more subtle, stable characteristics of the disease such as phenotypes, endophenotypes or intermediate traits, associated with schizophrenia. To allow better diagnostic procedures, therapeutic strategies and prediction of therapies [13], the use biomarkers that can be measured or determined from the easily available body fluids such as plasma/serum, urine or cerebrospinal fluid (CSF) is suggested (Figure 1). Biomarkers have three general functions: to improve diagnostic procedure as diagnostic biomarkers, to predict development of the disease as prognostic biomarkers (predisposition screening), and to be used in therapy monitoring as potential predictors of good or poor therapeutic response as theranostic biomarkers [13,14]. Biomarkers should be used in complex psychiatric disorders such as schizophrenia, since their use could facilitate diagnosis, prognosis (risk), prediction of disease, identification of specific symptoms, or pathological behaviors such as suicidal/aggressive/violent behavior, treatment response, and development of adverse events [7,13,15,16,17].

To facilitate deeper understanding of the molecular, cellular and systems alterations in schizophrenia, the use of diagnostic, prognostic but also theranostic biomarkers (i.e., novel tests or indicators or biomarkers used to identify the individual patient who will benefit from the most appropriate, efficacious treatment, given in the optimal dose, without toxic side effects and predict treatment response) is strongly recommended [13,16,17,18]. The term “theranostics” is defined as an answer to the unmet needs in medicine and in psychiatry to achieve personalized medicine approach in treatment; to improve diagnoses and to combine diagnostic processes with the best possible therapeutic strategies [18].

**Figure 2:**
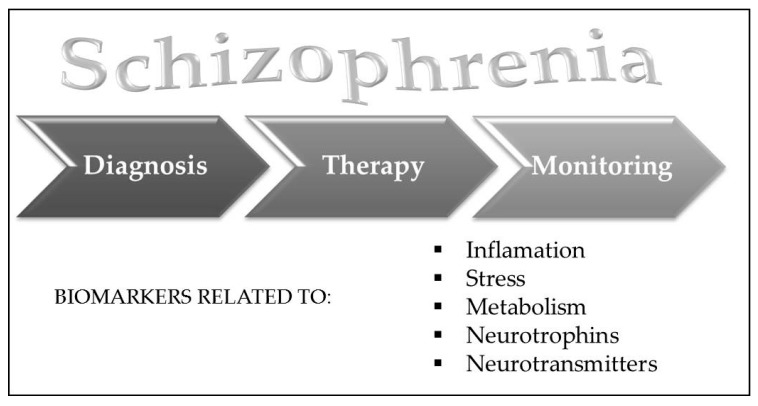
Diagnostic, prognostic and theranostic biomarkers for schizophrenia might be divided into inflammatory, stress-related, metabolic, neurotrophic and neurotransmitter biomarkers.

Biomarkers of schizophrenia may be divided into peripheral and central biomarkers. However, since schizophrenia is a disease of systemic nature, some biomarkers (i.e., analytes from the post mortem brains of patients with schizophrenia) are related to changes found in the blood, suggesting that brain/CNS and periphery are interconnected and therefore blood-based biomarkers are useful tools to reveal some processes in the brain [19]. These findings were recently confirmed and reviewed, since alterations in gene expression and epigenetic changes, together with alterations in proteomic and metabolic markers are detected in the CNS as well as in periphery [17]. This was explained by the reciprocal interactions that exist between the brain and the periphery, modulated by the neurotransmitters, hormones and cytokines, which affect gene expression of the peripheral indicators. On the other hand, the same molecules (hormones, neurotransmitters, or cytokines) might modulate central i.e., brain functions [17]. Biomarkers might be divided into those associated with the neuroimmune system (inflammatory biomarkers), neuroendocrine system (neuroendocrine related biomarkers), metabolism (metabolic biomarkers), neurotrophic factors (neurotrophic biomarkers), and neurotransmitters (neurotransmitter biomarkers), shown in Figure 2 [17].

### 3.1. Inflammation Biomarkers in Schizophrenia

Severe infectious illnesses are often accompanied by specific symptoms of schizophrenia, such as psychotic symptoms, mood disturbance and cognitive dysfunction [20]. A significant amount of evidence suggests that inflammation is a contributing factor to the pathophysiology of schizophrenia, and in agreement, changes in immunological markers have been found in blood, such as C-reactive protein (CRP) or different interleukins (IL) of patients with schizophrenia [21,22]. In addition, there are also data showing an association of different perinatal inflammatory cytokines with the development of schizophrenia later in life [23]. Some epidemiologic studies imply a connection between increased risk of schizophrenia and severe infection or autoimmune disease in anamnesis [24]. Besides, several genome-wide association studies (GWAS) emphasize a possible involvement of the major histocompatibility complex (MHC) locus and other immune regulatory regions in schizophrenia susceptibility [25].

CRP, as an acute-phase marker of inflammation, is found in higher concentrations in patients with schizophrenia than in healthy control subjects [26,27]. More specifically, higher CRP levels are shown to be associated with the severity of schizophrenia symptoms evaluated by the Positive and Negative Syndrome Scale (PANSS) [28,29]. Since elevated CRP levels are in correlation with an increased risk of metabolic syndrome in schizophrenia [30], there is an unmet need for researchers to control their results for possible confounding factors such as body mass index (BMI), gender or smoking. Perhaps this could be one of the explanations for inconsistencies between studies reporting elevated serum CRP levels in patients with schizophrenia and those reporting no association between CRP levels and schizophrenia [31]. CRP levels were determined in patients with chronic schizophrenia treated with different types of antipsychotics and switched to olanzapine treatment [32]. An increase of serum CRP concentration after 8 weeks of olanzapine treatment was reported [32].

Other potential markers of schizophrenia are different types of cytokines, proteins that modulate inflammatory processes and have an important role in development of CNS. A meta-analysis included 14 studies dealing with cytokine concentrations in serum of the first episode schizophrenia patients and reported increased concentration of interleukin IL-1β, IL-6, IL-12, tumor necrosis factor (TNF)-α, transforming growth factor (TGF)-β, interferon (IFN)-γ and soluble IL-2-receptor (sIL-2R) [33]. The same study suggested IL-1β, IL-6 and TGF-β as schizophrenia state markers, since they were elevated in both the first episode and the relapse of the disease, but these markers were decreased after antipsychotic treatment [33]. On the other hand, IL-12, IFN-γ, TNF-α and sIL-2R were presented as potential trait markers that do not normalize after the treatment [33]. Some other interleukins, such as IL-1RA, IL-10 and IL-15 [34], were also found to be increased in schizophrenia, while IL-6R, cluster of differentiation 5-like protein (CD5L), also known as apoptosis inhibitor expressed by macrophages, and IL-17 were even proposed to be a differentiating factor between pre-onset schizophrenia and bipolar disorder [35]. Besides, tumor growth factor (TGF)-α, CD5L, cluster of differentiation 40 (CD40), macrophage-derived chemokine and TNF receptor like 2 protein showed potential as predicting markers of schizophrenia relapse [36].

Interleukins and their role in the etiology of schizophrenia are investigated through the observed association between infections during pregnancy and risk for schizophrenia among offspring. Rather broad literature data, although inconsistent, indicate a higher risk of schizophrenia in people whose mothers were infected during pregnancy with influenza [37], rubella [38], polio [39], measles [40], herpes simplex virus-type 2 [41], or *Toxoplasma gondii* (toxoplasmosis) [42]. Since placenta acts as impermeable barrier for most infections, it is assumed that negative outcomes of maternal infections are caused by maternal and fetal responses to infection, which are primarily mediated by cytokines [43]. For example, there are studies reporting elevated levels of TNF-α and IL-8 in perinatal period of adult patients with schizophrenia [44,45]. More precisely, fetal exposure to increases in maternal IL-8 was found to be significantly associated with increased ventricular cerebrospinal fluid volume [46], a most commonly found brain disturbance in schizophrenia [47].

Schizophrenia shares similarities with some autoimmune diseases. In patients with schizophrenia, there are reports on increased levels of different auto-antibodies such as lupus anticoagulants, rheumatoid factors and antibodies against cardiolipin, *N*-methyl-D-aspartate (NMDA) receptor, DNA and dopamine receptor, with anti-NMDA receptor antibodies as the differentiating factor between schizophrenia and bipolar disorder [48]. Additionally, typical schizophrenia symptoms, cognitive deficits and acute psychosis could be found in autoimmune diseases, such as systemic lupus erythematosus [49]. Studies of autoantibodies in schizophrenia will hopefully contribute to understanding the neurobiological basis of disorder and findings of new treatment solutions.

Genetic studies estimate approximately 50% contribution of genetics to schizophrenia [50]. GWAS revealed a possible involvement of MHC region in genetic background of schizophrenia [51]. The MHC region consists of over 200 genes, including the TNF superfamily, human leukocyte antigen genes and complement cascade genes, and has an extremely important role in the regulation of immune system [52]. Further, GWAS covered five major psychiatric disorders (autism spectrum disorder, attention deficit-hyperactivity disorder, bipolar disorder, major depressive disorder, and schizophrenia) and reported that particular polymorphisms (for example rs2021722) within the MHC region are specific only for schizophrenia [53]. Additionally, alternate expression rates of the class I major histocompatibility complex genes in postmortem hippocampus samples of patients with schizophrenia were reported [54].

There are indications about the more effective treatment of schizophrenia symptoms with antipsychotics combined with nonsteroidal anti-inflammatory drugs (NSAIDs) [55] or aspirin as adjuvant therapy [56]. A study on mouse model of schizophrenia reported an attenuated amphetamine-induced behavioral impairment in mice treated with risperidone in combination with the NSAID celecoxib [57]. Additionally, in patients with first-episode psychosis, strong inflammation partly interfered with the effectiveness of antipsychotic drugs, with higher levels of IL-6 and IFN-γ associated with worse response to antipsychotic treatment [58]. In patients with schizophrenia seropositive for *Toxoplasma gondii*, treatment with antipsychotics with anti-toxoplasmic activity showed better results than treatment with antipsychotics without anti-toxoplasmic activity [59]. Meta-analysis including 23 follow-up studies found an increase of sIL-2 and decrease of IL-1β levels and IFN-γ after the treatment with antipsychotics, concluding that antipsychotic drugs show anti-inflammatory effects in schizophrenia [60]. Similarly, IL-1β, IL-6 and TNFα levels in serum of drug-naïve patients with first-episode psychosis were reduced after risperidone treatment [61].

Based on the current findings, the conclusion is that although predictive value of inflammatory biomarkers is still not completely defined, they still show great potential. Besides, these new findings represent a great contribution to some promising new therapeutic approaches such as biological immunotherapy in schizophrenia.

### 3.2. Neuroendocrine-Related Biomarkers in Schizophrenia

#### 3.2.1. The HPA Axis-Related Biomarkers in Schizophrenia

The HPA axis is important in development of schizophrenia since the HPA axis and sympatho-adrenal medullary system both modulate response to stress, which is one of the factors associated with its etiology. Besides stress, inflammation also significantly affects the HPA axis. Biomarkers in schizophrenia are related to hormones of the neuroendocrine systems, and also hormonal systems regulating metabolic system [14]. The HPA axis abnormalities and dysfunction are well recognized in schizophrenia, offering potential biomarkers, since patients with schizophrenia usually have elevated basal cortisol levels compared to values in healthy subjects [62,63,64,65,66,67,68]. These findings, showing either no effect or a small-to-medium increase in morning cortisol levels in patients with schizophrenia compared to controls, were confirmed in the recent meta-analyses [66,69]. These controversial results might be explained by the presence of various symptoms or different phases of schizophrenia [70]. Besides basal levels, patients with schizophrenia usually show altered response to challenge tests such as dexamethasone suppression test (DST) [62,63,65], CRH test [71], or DST/CRH test [72]. Namely, these tests assess the negative feedback of the HPA axis after administration of a challenge, and cortisol escape from suppression after DST is frequently seen in these patients [63,65]. DST non-suppression ranges from 0 to 81% [70]. In addition, HPA abnormalities are documented also by the findings showing glucocorticoid receptor downregulation associated with altered and reduced HPA axis system negative feedback mechanism, and increased cortisol secretion frequently related to reduced hippocampal volume in schizophrenia [10,11].

However, these data are not in harmony, and the results from the studies show both HPA hyper- and hypofunction in schizophrenia [70]. Patients with schizophrenia usually show diminished cortisol awakening response (CAR), i.e., cortisol concentration immediately after awakening [64]. In addition to morning alterations, diurnal rhythm of cortisol secretion is altered in the afternoon and evening in subjects with schizophrenia [73]. However, contradictory results can also be found [3,66,74]. Some recent reviews and meta-analyses were published to solve these contradictions: a meta-analysis of the 44 studies (*n* = 2613) showed only a moderate increase in morning cortisol levels in patients with schizophrenia compared to controls [66]. In a systemic review, in drug-naïve first-episode patients, elevated cortisol secretion was detected [70]. In patients receiving antipsychotic medication, atypical antipsychotic treatment (i.e., olanzapine, quetiapine and clozapine) usually decreases ACTH and cortisol levels [70]. We have previously reported that the typical antipsychotic fluphenazine increases, while the atypical antipsychotic olanzapine decreases, cortisol levels in patients with schizophrenia [75]. Therefore, a meta-analysis including a large group of medicated patients with schizophrenia (*n* = 1328) concluded that cortisol levels did not differ from the values in control subjects, while in medicated patients, most frequently treated with typical antipsychotics, cortisol is slightly increased compared to values in healthy subjects [66]. These results suggest that careful consideration of present medication use, type and doses is needed in the studies of the HPA axis biomarkers in schizophrenia. To exclude these confounding factors, first-episode patients or drug-naïve patients should be used. However, due to the complicated clinical picture of schizophrenia, biomarkers should be used also in patients with a chronic course of schizophrenia. Therefore, abnormal response of the HPA axis in schizophrenia is present, but cortisol is affected by numerous factors, such as body fluid (saliva or blood), time of sampling, stage of the illness, various symptoms, antipsychotic medication and stress induced by psychological and other environmental stressors [66,70]. Besides these factors, cortisol is under the influence of development and age [11]. Therefore, components of the HPA axis such as moderately increased cortisol, non-suppression to DST, altered diurnal rhythm of cortisol, and blunted or altered cortisol response to physiological stressors might be used as neuroendocrine diagnostic biomarkers of patients with schizophrenia, but should be compared to either drug-naïve patients or patients with a first episode of psychosis [70].

Genetic studies in neuroendocrine biomarkers of schizophrenia target most frequently two genes, and their single nucleotide polymorphisms (SNPs): a gene for FK506 binding protein-5 (*FKBP5*), encoding the protein FKBP51, and a gene for corticotrophin-releasing hormone receptor 1 (*CRHR1*), encoding the CRH type 1 receptors. FKBP5 is a protein (i.e., a co-chaperone) important in the stress response, and its genetic variants are involved in stress response since they affect glucocorticoid receptor (GR) function and GR complex and therefore modulate negative feedback. A risk variant of the *FKBP5* associated with higher FKBP5 induction may provoke prolonged cortisol release after stress, since it impairs the binding of cortisol to GR complex and inhibits its affinity to GR, decreases translocation, and impairs the negative feedback mechanism, leading to different psychopathologies and personality traits, altered responses to stress, disrupted homeostasis, epigenetic changes (*FKBP5* SNP rs1360780), and changes in the neural pathways, brain function and synaptic plasticity [1,76]. The rs1360780 risk allele of the *FKBP5* influences different regions of the brain associated with response to fear, threat and stress (amygdala and hippocampus), and in combination with exposure to early traumatic experience affects the amygdala and other brain regions connected to reactivity, emotional memory, and emotion processing. All these changes are associated with impaired reactions to threat and activated HPA axis. This particular gene x environment interaction induces altered responses to fear and stress associated with different psychopathological phenotypes [1,76]. FKBP5 overexpression was found in different regions of the postmortem brains, which are associated with Alzheimer’s disease and schizophrenia [76]. Different SNPs of the *FKBP5* and their risk genotypes (rs3800373, rs9296158, rs1360780, and rs9470080) interacted with early traumatic experiences and were associated with aggressive and violent behaviors [77]. *FKBP5* SNPs (rs9296158, rs1360780, rs1043805, and rs4713916) were also found to interact with childhood trauma to predispose to development of psychotic symptoms [78]. Besides these aberrant behaviors frequent in psychiatric disorders, FKBP5 was reported to be increased in human adipose tissue after dexamethasone, implicating its role in metabolic disorders such as insulin resistance [79]. These results all suggest that FKBP5 disinhibition might be used to characterize endophenotypes sensitive to stress, associated with various stress-related disorders, and therefore the blockade of the FKBP5 might be used in the treatment of different phenotypes induced by stress [76].

Another genetic component of the HPA axis that is frequently investigated is a gene for *CRHR1*. These CRHR1 regulate HPA axis activity and its negative feedback, and therefore its genetic variants modulate CRH transmission, and under other environmental factors such as childhood trauma may induce changes in CRHR1 signaling and HPA axis disruption, and altered stress response [1]. Gene coding for the CRH binding protein (*CRHBP*) was investigated in schizophrenia. The heterozygous genotype of the *CRHB* (rs1875999) was significantly associated with suicidal attempts, while the rare variant of BclI polymorphism in the glucocorticoid receptor gene (*NC3R1*) 11 was associated with protection from suicidal attempt in schizophrenic patients [80]. In addition, the interaction between the two genes related to HPA axis, *CRHR1* and *CRHBP* was detected in patients who attempted suicide and with severity of suicidal behavior in schizophrenia [80]. The association between *CRHR1* and *CRHBP* genes with severity of suicidal behavior was found in patients with schizophrenia [80]. All these genetic studies point to the importance of the gene x environment interactions, and suggest that genes coding for proteins important in the regulation of the HPA axis and stress response may contribute to long-lasting changes in the HPA axis function, and may alter and disrupt negative feedback mechanism, homeostasis and emotion regulation, leading to different mental disorders and altered behaviors [1].

#### 3.2.2. Metabolic Biomarkers

Somatic disorders such as metabolic disorders as well as metabolic syndrome, type 2 diabetes, insulin resistance and different cardiovascular diseases are frequent in schizophrenia [67,81]. Patients with schizophrenia have increased risk of having higher than normal body mass index (BMI), being smokers, having increased glucose levels, and developing diabetes, hypertension and dyslipidemia, all risk factors for cardiovascular disease, compared to healthy people [82]. Expression of various hormones was found to differ significantly between patients with schizophrenia and control subjects: insulin, prolactin, pancreatic polypeptide, progesterone and chromogranin A (a protein of the granin family composed of 439 amino acids that is located in the large dense-core vesicles of the neuroendocrine cells) were increased, while growth hormone was decreased in patients with schizophrenia compared to control subjects [67]. These results might explain how chronically elevated insulin levels affect brain function and contribute to neuroinflammation, altered phosphorylation and promote deposition of amyloid plaques, leading to changes characteristic of dementia [14]. Neuroendocrine biomarkers, such as cortisol, insulin, leptin, pro-opiomelanocortin, prolactin and growth hormone were similarly altered in the brain and in the blood in patients with schizophrenia [19]. Other neuroendocrine biomarkers altered in schizophrenia are gonadal hormones estradiol and testosterone, suggesting gender differences in schizophrenia [14], as well as hormones of the thyroid gland such as decreased thyroxine, tri-iodothyronine, and thyroid-stimulating hormone [83], suggesting that these biomarkers might represent peripheral plasma markers of oxidative stress and thyroid dysfunction in schizophrenia.

There are controversial findings of the levels of the neurosteroids dehydroepiandrosterone (DHEA) and DHEA sulphate (DHEAS), collectively known as DHEA(S), in schizophrenia [84]. They have neuroprotective and neuromodulatory effects, and were reported to be increased, decreased or similar in schizophrenic patients compared to healthy control subjects [84]. In addition, there are also findings showing the augmentation strategies using DHEA(S) in the treatment of schizophrenia: in studies performed on a small number of patients, addition of DHEA(S) as the augmentation strategy improved somatic health, decreased insulin resistance and inflammatory markers and had positive effects on the quality of life or physical disability [84].

These results confirm that some indicators of metabolic syndrome or insulin resistance, or the hypothalamic–pituitary–adrenal–gonadal axis are present in patients with schizophrenia, and that these neuroendocrine and/or metabolic biomarkers might be used to discriminate between patients with or without the risk for metabolic disorder, improve the understanding of the pathophysiological processes associated with schizophrenia, advance the development of treatments focused on these endocrine and metabolic disorders and consequently help in personalized medicine strategies [14,67].

### 3.3. Neurotrophins as Candidate Biomarkers in Schizophrenia

#### 3.3.1. Brain-Derived Neurotrophic Factor

Neurotrophins play a significant role during the development of the nervous system by promoting neuronal growth and differentiation, but they are also very important in the adult brain since they modulate neuronal plasticity and function. Nerve growth factor (NGF), brain-derived neurotrophic factor (BDNF), neurotrophin (NT)-3 and NT-4/5 are the members of neurotrophin family which are located in the mammalian brain. All of these neurotrophins are synthetized as pro-neurotrophin precursors which are then proteolytically cleaved to yield mature proteins [85]. Binding to an appropriate tyrosine kinase receptor (Trk) or pan neurotrophin receptor (p75NTR) activates different signaling pathways leading to specific and diverse biological effects of neurotrophins [86]. Lately, neurotrophins and their precursors have aroused great interest as the possible players in the pathophysiology of several psychiatric disorders, including schizophrenia. According to the neurodevelopmental hypothesis, schizophrenia is considered to be the result of pathologic processes that began during pre- and post-natal development of the CNS [87]. Since BDNF has an important role in the processes of CNS development and maintenance, it is considered to be one of the main players in light of the neurodevelopmental hypothesis.

Studies have shown significantly reduced expression of *BDNF* gene, gene coding for TrkB receptor, and significantly decreased BDNF protein concentration in the hippocampus of patients with schizophrenia [88,89]. However, a study by Durany and colleagues further pointed out an increased BDNF concentration in cortex of individuals with schizophrenia [89]. Meanwhile, there is also evidence of lower BDNF levels in prefrontal cortex and in CSF of subjects diagnosed with schizophrenia [90]. In the last decade, many research strategies have focused on the peripheral BDNF as a potential biomarker in psychiatry. The sources of the peripheral BDNF are platelets and immune and vascular endothelial cells [91,92], but there are also indications that BDNF is able to cross the blood–brain barrier [93]. Studies of BDNF as a peripheral biomarker in psychiatric disorders, including schizophrenia [94], are supported by a strong positive correlation between peripheral BDNF levels and BDNF levels in the CNS [95,96]. Low serum BDNF protein levels were determined in drug-naïve patients with schizophrenia [97,98,99,100], but also in schizophrenic patients who were treated with different antipsychotics [101,102,103,104,105]. These results were confirmed by a meta-analysis, which included 16 individual studies [106]. A significant correlation between serum BDNF levels and clozapine daily dose was found, suggesting also that the treatment with clozapine can lead to cognitive enhancement in patients with schizophrenia [107]. Another study, which drew attention to the existence of three different isoforms of BDNF in the serum, (i.e., precursor, mature and truncated forms of BDNF), suggested that individuals with schizophrenia have lower levels of truncated isoform, compared to other forms of BDNF [108]. The biological role of the truncated isoform of BDNF has not yet been clarified. Animal studies demonstrated a significant effect of antipsychotic treatment on BDNF brain levels. Treatment with haloperidol decreased *BDNF* messenger RNA (mRNA) and protein levels in different brain areas, including hippocampus and prefrontal cortex [109,110,111], and a similar effect was observed for risperidone administration [109]. In addition, a switch from haloperidol, which negatively affects the expression of BDNF, to olanzapine treatment, restored BDNF levels to normal values [112]. Findings by Chlan-Fourney and colleagues demonstrated that hippocampal BDNF mRNA expression is affected by chronic, but not acute treatment with antipsychotics [113]. The observed effect was most likely a result of long-term changes in gene regulation, rather than blockade of the serotonergic receptors type 2 (5-HTR2) and dopaminergic receptors type 2 (DRD2). Even though existing studies have failed to irrefutably prove the effect of treatment with antipsychotics on peripheral BDNF concentration and mRNA expression, there is a need for additional investigations, which will help to further clarify the role of neurotrophins in the response to treatment with different atypical antipsychotics.

The association studies between genetic polymorphisms of *BDNF* and schizophrenia resulted in contradictory findings. Literature search usually yields results concerning two of the most common SNPs of the *BDNF* gene, Val66Met (rs6265) and C270T (rs56164415). Polymorphism Val66Met is functional since it affects the BDNF mRNA intracellular trafficking and the activity-dependent secretion of BDNF protein by disturbing the interaction of BDNF with transport molecules like sortilin [114,115]. The C270T polymorphism is assumed to modulate the *BDNF* gene expression and post-transcriptional regulation of this expression, due to its position in the 5′ untranslated region and the assumptions that it is located within CpG islands [116]. Some studies have shown that the *BDNF* Met allele or the Met/Met genotype is associated with the increased risk of developing schizophrenia [117,118]. However, it was also reported that the *BDNF* Val allele could be related to lower hippocampal volume [119] and be one of the genetic risk factors for schizophrenia [120,121]. Most of the studies linking *BDNF* Val66Met to schizophrenia found no association between this polymorphism and development of schizophrenia [122,123,124,125,126]. Even though a meta-analysis conducted by Zintzaras found no evidence for the association between *BDNF* Val66Met polymorphism and schizophrenia, it suggested a possible association between schizophrenia and the other SNP, *BDNF* C270T polymorphism [126]. This association was confirmed in the East Asian population by another meta-analysis based on 13 case-control association studies [127], suggesting that the T allele may contribute to predisposition to schizophrenia. Some other authors also observed a higher frequency of the T allele carriers and C/T heterozygotes in subjects with schizophrenia, compared to healthy individuals [128,129]. However, there are studies that did not confirm these results [123,124,130]. Genetic variants of *BDNF* were also reported to be significantly associated with response to treatment with antipsychotics. Few studies reported association between *BDNF* Val66Met polymorphism and treatment response in schizophrenic patients, indicating a higher frequency of the Val/Val homozygotes in clozapine treatment responders [131,132], and olanzapine treatment responders [133], compared to non-responders. However, there are studies that failed to confirm the above mentioned associations between the *BDNF* Val66Met polymorphism and clozapine treatment response [134,135]. Study by Zai and colleagues also suggested an over-representation of the rs11030104 T/T genotype and the T allele in responders compared to non-responders, and an association between the marker rs1519480 and antipsychotic-induced weight gain [132]. Another genetic *BDNF* marker, dinucleotide microsatellite repeat polymorphism (GT)n, was associated with treatment response and chlorpromazine-induced extrapyramidal reverse effects in Chinese patients with schizophrenia [136]. Other studies have linked antipsychotic-induced weight gain with *BDNF* genetic variants, including *BDNF* Val66Met polymorphism [137], rs11030101 [138], and rs1519480 [132]. A systematic review and a meta-analysis by Cargnin and colleagues, which included nine different studies, found no evidence that would irrefutably support the involvement of *BDNF* gene variants in the antipsychotic drug response [139]. However, these authors suggested that future studies should focus on haplotype combinations, which include *BDNF* Val66Met polymorphism with other *BDNF* SNPs.

Different epigenetic mechanisms are responsible for activity-dependent regulation of *BDNF* gene expression [140,141], implicating the possible role of the regulation of *BDNF* expression in several psychiatric disorders including schizophrenia [142,143,144]. There are only a few studies that investigated the promoter methylation frequency in patients with schizophrenia. Mill and colleagues found no difference in DNA methylation profiles between patients with schizophrenia and bipolar disorder, compared to healthy controls [145]. A study by Igekame and colleagues revealed the hypermethylation of one CpG site in the *BDNF* promoter I and no difference in the *BDNF* promoter IV methylation frequency between schizophrenic patients and healthy individuals [146]. However, the results from the other study suggested a difference in the promotor IV methylation frequency and *BDNF* gene expression between patients diagnosed with schizophrenia and control subjects [147]. Yet the question remains as to whether the alterations in methylation status of specific genes are directly associated with the pathophysiology of schizophrenia, or are a consequence, at least in part, of an antipsychotic treatment. Global DNA hypomethylation was reported in the leukocytes of patients diagnosed with schizophrenia [148], which could be related to the chronic treatment with different antipsychotics demonstrating DNA-demethylation activity [149].

#### 3.3.2. Other Neurotrophins

There are only few studies that have addressed the role of other neurotrophins in pathophysiology of schizophrenia. Decreased NT-3 levels in the cortex of schizophrenia patients compared to non-psychotic individuals were detected [89]. Different gene association studies suggested an association between dinucleotide repeat polymorphism in the promoter region of gene coding for NT-3 and schizophrenia [150,151], but these results were not confirmed [152]. However, the carriers of A3/147-bp allele in a dinucleotide repeat polymorphism were reported to have earlier age of onset and more serious extrapyramidal symptoms [153]. Another *NT-3* polymorphism, Gly63Glu, which results in an amino acid substitution of glycine by glutamic acid at position 63, has also been associated with schizophrenia, earlier age of onset and the duration of illness [154].

A few recent studies demonstrated reduced NGF blood levels in the first-episode schizophrenia patients compared to healthy individuals [155,156]. Decrease in NGF and NGF receptor (NGFR) blood levels was confirmed in drug-naïve schizophrenia patients and patients treated with haloperidol [157]. In addition, an association between different *NGF* (rs6330, rs4839435) and *NGFR* (rrs11466155, rs2072446, rs734194) polymorphisms and schizophrenia were also found [157].

There is convincing evidence of an association between BDNF and schizophrenia. Even though changes in the mRNA expression and protein concentration of BDNF and other neurotrophins in the brain and at the periphery are not specific only for schizophrenia patients, further studies are needed to determine if these systemic alterations are the cause or consequence of the neurodevelopmental changes in schizophrenia.

### 3.4. Neurotransmitter Biomarkers

#### 3.4.1. Dopaminergic System

Various findings demonstrated the hyperfunction of dopaminergic system in the mesolimbic system of patients with schizophrenia creating the “dopamine (DA) hypothesis of schizophrenia” [158,159,160,161,162]. This classical DA hypothesis of schizophrenia was mostly concerned with the hyperactivity of DA signaling in subcortical regions such as striatum [162,163,164,165,166], and with significant role of DRD2 in the development of positive symptoms [167,168,169,170]. This dopaminergic hypothesis of schizophrenia has been also supported by the correlation between efficacy of antipsychotic drugs in treating schizophrenia and their potency to antagonize the binding of DA to DRD2 [171,172,173], as well as by the ability of dopaminergic agents, such as psychostimulant amphetamine, to induce excessive release of striatal DA and stimulate schizophrenia-like psychosis [174,175,176]. However, enduring negative and cognitive symptoms of schizophrenia, resistant to DRD2 antagonist treatment, suggested that they might be due to a deficit in the prefrontal cortex (PFC) DA transmission at DRD1 [177,178,179,180]. According to this hypothesis of cortical/subcortical imbalance in schizophrenia, hypoactive DA neurotransmission in PFC, resulting in hypostimulation of DRD1, and negative and cognitive symptoms, leads to disinhibition of subcortical mesolimbic DA activity, resulting in hyperstimulation of DRD2 and positive symptoms of schizophrenia [159,181,182,183]. Therefore, the optimal schizophrenia treatment should increase DA activity in the mesocortical regions and decrease mesolimbic DA activity [184]. These findings also stimulated further research investigating the risk genes involved in the pathophysiology of schizophrenia [185]. These genetic studies also included the polymorphisms in the genes coding for various DA receptors and their association with schizophrenia [186,187,188,189], as well as with antipsychotic treatment response [190,191,192,193], however with contradictory results. Various markers of dopaminergic function have been also investigated in the blood of subjects with schizophrenia [194], in order to find peripheral biomarkers. For instance, DA uptake by platelets was shown to correlate with delusional state in subjects with schizophrenia [195]. Tyrosine hydroxylase (TH), the enzyme involved in the synthesis of DA, has been reported to be increased in lymphocytes of schizophrenia patients [196]. Although elevated lymphocyte mRNA levels of DA transporter (DAT) were demonstrated in patients with schizophrenia [197], reduced density of the lymphocyte DAT proteins was reported in psychosis [198]. The plasma or CSF levels of homovanillic acid (HVA), the principal DA metabolite, were found to be elevated in schizophrenia [199,200,201,202], and to predict response to antipsychotics drugs [203,204,205]. Some authors reported the up-regulation of *DRD2* mRNA expression and DRD2 binding in lymphocytes from schizophrenia patients [206,207], although other studies have not confirmed these findings [197,208]. In addition, *DRD2* mRNA levels in lymphocytes are suggested to correlate with positive symptoms of schizophrenia [197], whereas lymphocyte DRD2 binding was reduced after treatment with antipsychotic drug loxapine [209]. The results regarding DRD3 were also conflicting, demonstrating increased [208,210,211], as well as decreased [212] levels of lymphocyte *DRD3* mRNA in schizophrenia. *DRD4* mRNA in lymphocytes was found to be lower [208], or un-changed [197] in patients with schizophrenia compared to control subjects. The changes in lymphocyte *DRD3* and *DRD5* mRNA seem to be associated with schizophrenia symptom severity [211], with increased levels observed following antipsychotic treatment [211,212]. Moreover, some studies demonstrated the increased binding of DA antagonists in lymphocytes of subjects with schizophrenia in comparison to control group [206,213], as well as in responders to antipsychotic treatment as compared with treatment-resistant schizophrenia patients [214].

Genes associated with schizophrenia and antipsychotic drug response include the catechol-*O*-methyl transferase (*COMT*) gene, coding for an enzyme involved in the catabolism and regulation of DA levels in the PFC [215,216]. Various studies linked schizophrenia with a region on chromosome 22q11.2, where the *COMT* gene has been located [217,218]. Many association studies investigated Val158/108Met (rs4680) functional polymorphism in the *COMT* gene. The Val/Val genotype of this polymorphism, which results in the higher COMT activity compared to Met/Met genotype, may lead to a decreased DA neurotransmission in PFC [219,220], characteristic for schizophrenia [221]. However, the studies yielded inconsistent results, associating both the high activity “Val”, and low activity “Met” alleles with schizophrenia [215,222,223,224,225,226], or failing to detect any association [227,228]. The more recent meta-analysis found only a weak association of this polymorphism with schizophrenia [229]. Inconsistent associations of Val158/108Met in subjects of Asian origin have been attributed to another *COMT* gene polymorphism, Ala72Ser (rs6267) [230]. Other reports also demonstrated the association of additional *COMT* polymorphisms and haplotypes with schizophrenia [231,232,233,234], as well as the synergistic effects of *COMT* gene with genes involved in other neurotransmitter systems [235,236]. In addition, the hypomethylation of the *COMT* gene promoter, which leads to over-expression of COMT, has been found in post-mortem brains [237], as well as in peripheral blood cells of schizophrenia patients [148,238,239]. As COMT hyperactivity in schizophrenia might result in decreased dopaminergic neurotransmission in the PFC, and consequently to the development of negative and cognitive symptoms of schizophrenia [215], different COMT inhibitors have been investigated for potential treatment [240,241,242,243,244,245,246]. It has been demonstrated that COMT inhibition potentiates the increase in extracellular DA in PFC elicited by the antipsychotic drug clozapine [247]. Regarding antipsychotic response, the findings of the studies investigating the role of *COMT* Val158/108Met polymorphism were also conflicting, showing that schizophrenia patients with Met/Met genotype have poor [248], as well as good response to antipsychotic treatment [233,249,250,251,252,253]. However, some authors found no association of this polymorphism with the therapeutic response to antipsychotic drugs [254,255,256]. In addition, higher daily antipsychotic doses seem to be needed in schizophrenia patients, homozygous for the Met allele [257,258]. Other polymorphisms and haplotypes in the *COMT* gene have been also associated with response to antipsychotic treatment [233,251,259].

#### 3.4.2. Serotonergic System

The involvement of the serotonergic system in schizophrenia was suggested from the studies demonstrating that lysergic acid diethylamide (LSD), structurally similar to serotonin (5-HT), produces various schizophrenia-like symptoms [260], as well as from the ability of some atypical antipsychotics, such as clozapine, to improve symptoms of schizophrenia by modulating the 5-HT concentration [261]. It has been suggested that dopaminergic hypofunction, observed in schizophrenia, might be due to the up-regulation of ascending serotonergic pathways [262]. The disturbed interconnectivity between serotonergic and dopaminergic [263], as well as between serotonergic, cholinergic and gamma-amino-butyric acid (GABA)ergic systems in the cortex and hippocampus [264,265], might play an important role in the complex pathophysiology of schizophrenia [266]. The decrease in the density of the 5-HT transporter (5-HTT), increase in 5-HTR1A number and reduction of 5-HTR2 levels in the brain have been most frequently reported evidence of the involvement of serotonergic system in schizophrenia [260]. Various 5-HT receptors have been particularly associated with cognitive impairment in schizophrenia [267]. Reports from post-mortem studies have shown reduction in the 5-HTT number [268,269]. Moreover, a recent meta-analysis reported increased 5-HTR1A binding in PFC, but diminished or unchanged in the amygdala [270]. Since stimulation of 5-HTR1A was found to promote the release of dopamine in PFC, it is possible that the effects of antipsychotic drugs such as clozapine, ziprasidone and aripiprazole are partly achieved by their agonistic action via 5-HTR1A [271]. Increased hippocampal *5-HTR1B* mRNA levels were also observed in subjects with schizophrenia [272]. In spite of the conflicting results obtained in vivo [273,274], reduced density of prefrontal 5-HTR2A has been found in the post-mortem studies of schizophrenia [270]. Concerted 5-HTR1B up-regulation and 5-HTR2A down-regulation could result in decreased GABAergic and increased glutamatergic activity in the hippocampus of schizophrenia patients [272]. Although no changes in the number of 5-HTR3 and 5-HTR4 were found in the brain of schizophrenia patients [275,276], the associations of *5-HTR3E* and *5-HTR4* gene variants with schizophrenia have been reported [277,278]. Various studies also supported the role of 5-HTR7 in the neurobiological basis in schizophrenia, including reduction in the 5-HTR7 expression in the hippocampus and prefrontal cortex [279,280], the association of *5-HTR7* gene haplotype with development of schizophrenia [281], the affinity of some antipsychotic drugs for 5-HTR7 [279,282], and the up-regulation of 5-HTR7 following antipsychotic treatment [279]. Studies measuring peripheral serotonergic biomarkers found contradictory results. Increased platelet 5-HT concentration has been demonstrated in schizophrenia patients [283,284,285]. However, lower baseline 5-HT concentrations in plasma and platelets were observed in poor responders to antipsychotic drugs in comparison to control subjects and increased significantly during treatment [286]. On the other hand, Kaneda and colleagues reported lower platelet 5-HT in the subjects with schizophrenia receiving antipsychotic treatment [287]. Potential association of genetic variations of 5-HT receptors and 5-HTT with schizophrenia and therapeutic response to antipsychotics has been intensively investigated in a large number of studies [288,289,290]. Genetic polymorphisms of *5-HTR2A* and *5-HTR2C*, at which act many antipsychotic drugs, have been the main targets of pharmacogenetic analyses [192,291,292]. However, some studies found no associations between polymorphisms in genes encoding 5-HT receptors and 5-HTT with antipsychotic treatment response [190,293]. Regarding the involvement of serotonergic system in the development of side effects of antipsychotics, pharmacogenetic research has focused primarily on weight gain [294,295,296,297], while relatively small number of studies has investigated the extrapyramidal side effects [298]. Although some authors reported the associations between tardive dyskinesia and *5-HTR2A* and *5-HTR2C* gene polymorphisms, the results are not uniform [299,300]. In addition to genetic research, some epigenetic studies have demonstrated DNA methylation changes such as hypermethylation of *HTR1A* gene [301], and differential methylation of the *HTR1E* gene [238] in peripheral blood cells of schizophrenia patients. Based on current findings, different authors suggested agents modulating 5-HTR2C [302,303], 5-HTR3 [304], and 5-HTR6 [305], as potential schizophrenia drug targets.

#### 3.4.3. Norepinephrine System

A number of studies supported the norepinephrine hypothesis of schizophrenia [306], suggesting that elevated norepinephrine signaling may be involved in the pathophysiology of schizophrenia [307,308,309,310]. Increased concentrations of 3-methoxy-4-hydroxyphenylglycol (MHPG), the metabolite of norepinephrine, were demonstrated in plasma of subjects with schizophrenia [205], but not in CSF [311]. On the other hand, decrease in plasma MHPG was observed during treatment to antipsychotic treatment [312,313]. However, antipsychotic drugs risperidone and clozapine have been shown to elevate plasma norepinephrine levels, whereas risperidone produced a smaller effect [314]. Therapeutic approaches using norepinephrine drugs, especially α-2A agonists, in combination with antipsychotics have been proposed for treatment of cognitive impairments in schizophrenia [315].

#### 3.4.4. Cholinergic System

The cholinergic system has been associated with schizophrenia in various reports [316], mainly suggesting down-regulation of cholinergic activity [317]. Decreased mRNA levels for α-7 acetylcholine receptors have been found in the post-mortem brain [318], as well as in lymphocytes of subjects with schizophrenia [319]. Post-mortem studies demonstrated lower α-7 nicotinic receptor binding in the hippocampus and thalamic nuclei [320,321], as well as reduced α-7 receptor protein expression in the frontal cortex of subjects with schizophrenia [322]. Disturbed cholinergic function, particularly involving nicotinic receptors, has been linked with higher smoking frequency among schizophrenia patients in comparison to general population [323]. Some studies also suggested a nicotinic cholinergic α-7 receptor as a promising target for therapy of cognitive deficits in schizophrenia [324,325]. An increasing body of evidence has also implicated muscarinic system as contributing to a number of symptoms of schizophrenia, especially related to cognitive impairment [326]. Down-regulation of muscarinic receptors has been observed in large areas of the post-mortem brain of patients with schizophrenia [327,328]. Allosteric modulation of the M1 muscarinic acetylcholine receptor has been proposed as a potential target for therapeutic interventions in schizophrenia [329].

#### 3.4.5. Glutamatergic System

Different schizophrenia susceptibility genes and various schizophrenia-related environmental factors influence glutamatergic neurotransmission [330,331,332,333,334]. However, one of the main findings supporting the glutamatergic hypothesis, based on the glutamate-dopamine dysbalance in schizophrenia, is that phencyclidine and ketamine produce psychosis, with positive, negative and cognitive symptoms, resembling those in subjects with schizophrenia [335,336,337]. Treatment with these *N*-methyl-d-aspartate receptor (NMDAR) antagonists induce schizophrenia-like dopaminergic dysregulation in human subjects [338], as well as in animal models [339]. Post-mortem studies have shown variable changes in the expression of NMDAR in the brain of schizophrenia patients [340,341,342]. On the other hand, some studies indicated greater sensitivity of NMDAR in patients with schizophrenia [343]. The involvement of abnormal glutamate transmission in various impaired neurophysiological measures, observed in schizophrenia, has been suggested by Javitt and colleagues [344]. In agreement with the proposed role of glutamatergic system in the pathophysiology of schizophrenia [173], many researchers investigated the peripheral levels or functions of various amino acids that activate glutamate receptors and modulate glutamatergic neurotransmission [343]. In addition to reduced markers of glutamatergic neuronal integrity in the plasma and CSF of schizophrenia patients [345], lower concentrations of glutamate [346], glycine [332,347], and d-serine were observed [348]. However, some studies demonstrated increased concentrations of glutamate [349], agmatine [350], glutamine [351], d-serine and glycine [352] in plasma or blood from subjects with schizophrenia, in comparison to control subjects. Lower plasma d-serine and d-/l-serine ratio were found in the treatment-resistant schizophrenia patients, whereas glycine levels and glycine/l-serine ratio were increased following clozapine treatment [353]. In addition, d-serine plasma levels were associated with improvements in positive [352], but not in cognitive symptoms of schizophrenia [354]. Agonists acting via glycine-site of NMDAR induced improvements in negative and cognitive symptoms, whereas d-serine and sarcosine, administered together with antipsychotics, reduced positive symptoms in patients with schizophrenia [355,356,357]. In addition to NMDAR, the potential glutamatergic drug targets for schizophrenia treatment include also metabotropic (mGluR2/3) glutamate receptors [358,359,360].

#### 3.4.6. GABAergic System

Various research evidence support abnormal GABAergic neurotransmission in schizophrenia [361,362,363,364,365,366,367]. One line of findings came from the post-mortem studies demonstrating a decrease in the density, as well as specific abnormalities of GABAergic interneurons, in the hippocampus and PFC of schizophrenia patients [362,366], regions important for the somatosensory information processing. The hypothesis of dysfunctional GABAergic interneurons is supported by the observed hypermethylation in cortex and basal ganglia of subjects with schizophrenia [368,369,370,371,372,373], resulting in a down-regulation of GABAergic genes and GABAergic neuronal circuit dysregulation [369,374,375]. Such hypermethylation has been observed also in peripheral blood lymphocytes from patients with schizophrenia [376]. One of such hypermethylated genes is the gene coding for glutamic acid decarboxylase (GAD), the rate-limiting enzyme in GABA biosynthesis, for which expression is found to be generally lower in post-mortem brain of schizophrenia patients compared to controls [361,377,378,379,380,381]. In accordance with this hypothesis of GABAergic hypofunction in schizophrenia, GABA_A_ receptor agonists might have beneficial effects when administered alone or in combination with antipsychotic drugs. Some of the other results suggesting GABAergic neuropathology in schizophrenia include lower GABA levels in plasma of subjects with schizophrenia [199], down-regulation of prefrontal GABA_A_ receptor α5 subunit mRNA [382], and the associations of different polymorphisms and haplotypes in the GABA_A_ receptor β2 subunit gene (*GABRB2*) with schizophrenia [383]. Moreover, the density of the peripheral-type benzodiazepine receptors in platelets is suggested as a possible predictor for aggressive behavior in schizophrenia patients [384].

## 4. Conclusions

Schizophrenia is a complex disease associated with different alterations in the brain circuits and molecular pathways, characterized with different signs and completely different symptoms [2]. This review described the potential of the use of specific markers associated with immune processes, or with metabolic disorders or neuroendocrine/neurotrophin/neurotransmitter alterations that might help in discrimination of patients with specific, different underlying pathology or treatment response. Better classification or definition of the more homogenous groups will improve understanding of the biological underpinning of schizophrenia, and consequently improve treatment strategies and personal medicine approaches. This was recently confirmed in first episode psychosis patients, where non-responders to treatment had significantly lower CAR and significantly higher IL-6 and IFN-γ levels than responders to treatment [58]. These data suggested that blunted CAR in combination with proinflammatory IL-6 and IFN-γ levels that were elevated in non-responders could be used as trait markers, and as predictors of the poor treatment response [58]. The combination of selected cytokines (sIL2—increased by antipsychotics, and IL-1β and IFN-γ—decreased by antipsychotics) could be used as marker of the treatment response, since antipsychotic treatment corrected the inflammation in schizophrenia [60].

The neurotrophin BDNF is an interesting candidate in the search for biomarkers that could improve diagnosis and therapy monitoring in patients with schizophrenia. Most of the studies reported reduced expression of *BDNF* gene and significantly decreased BDNF protein concentration in different brain areas, and decreased concentration of plasma BDNF in patients with schizophrenia [88,89,90,97,98,99,100]. In contrast to neuroendocrine and immune markers that were associated with treatment response, BDNF was confirmed to be significantly reduced in plasma samples of schizophrenia patients, either in medicated or in drug-naïve patients [106]. Chronic treatment with antipsychotics affected *BDNF* mRNA and protein expression in both the CNS and at the periphery [106]. Significant association was reported for *BDNF* Val66Met polymorphism and antipsychotic treatment response, showing that better therapeutic response was found in carriers of the Val/Val homozygous genotype and suggesting that this genotype might be used to predict good treatment response in schizophrenia [131,132,133]. Future studies should focus on linking specific *BDNF* haplotype combinations with treatment response in patients with schizophrenia [139].

However, confounding factors, such as sex, the use of oral contraceptive pills, or pre-and postmenopausal status or phases of the menstrual cycle should be controlled, since they present sources of variation that might significantly impact biomarker findings [385]. Besides these hormonal variables, biomarkers should be adjusted for age, BMI, medication, but also other demographic, lifestyle, and health variables [385]. Opposed to previous criteria [386] that included paranoid, disorganized, catatonic, residual and undifferentiated subtypes of schizophrenia, new diagnostic DSM‑5 criteria [387] removed schizophrenia subtypes as they were associated with poor reliability, low stability over time, and had insignificant prognostic value, and therefore did not contribute to improved treatment or better prediction of the treatment response [388]. Due to the reduced utility of schizophrenia subtypes and their poor diagnostic and prognostic values [388], this review did not focus on markers or the combinations of markers that could be best suited to differentiate between different forms or subtypes of schizophrenia.

At present, there are still no validated laboratory tests, biomarker(s) or a panel of combined markers for schizophrenia diagnosis, prognosis or the prediction of the treatment response. Selected serum analytes (among the 34 analytes betacellulin, bone morphogenic protein 6, eotaxin 3, follicle stimulating hormone and epidermal growth factor were significantly altered in first-onset antipsychotic naïve patients with schizophrenia compared to control subjects), were suggested to indicate a reproducible biological signature, but these studies need replication in larger population and in longitudinal studies [389].

Despite intensive research, due to many inconsistencies among studies, no neurotransmitter markers have been considered as valid theranostic biomarkers for schizophrenia which could soon enter the clinical routine [390]. In addition to the components of monoamine systems, other candidate neurotransmitter biomarkers recently received increased attention [391]. However, there is still a limited evidence of their validity and specificity in schizophrenia, as so far these findings have not been sufficiently reproduced in larger cohorts and independent samples [392]. Although neurotransmitter changes observed in the brain are often not accompanied with the same patterns in the periphery, evidence suggests that they can be used as potential biomarkers of schizophrenia treatment efficacy [17]. This refers especially for the peripheral monoamine-related molecules that, due to their strong association with the pharmacological properties of antipsychotic drugs, might represent good indicators of treatment response, instead of being stable trait markers suitable for straightforward diagnosis of schizophrenia [17].

Recently [393], schizophrenia was postulated to represent altered homeostasis of immune/inflammatory, oxidative stress, endocrine and metabolic signaling processes that mediate and affect dopaminergic, serotonergic, glutamatergic, GABA-ergic and cholinergic neurotransmission and white matter-associated neural connectivity. This review confirmed that neuronal connectivity is modulated by the cross-talk among molecular markers of these systems associated with disturbances in stress signaling, and vice versa [393]. Numerous molecular substrates, responsible for the imbalance in homeostatic signaling, should be used to discover validated biomarkers or biomarker test in schizophrenia.

Therefore, the future use of diagnostic, prognostic and theranostic biomarkers will improve diagnosis, therapy monitoring, and prediction of treatment outcome [13,14,15,16,17]. These biomarkers, when validated and approved for clinical practice, will improve the quality of life in patients with schizophrenia and decrease health costs worldwide.

## Abbreviations

HPA	Hypothalamic–pituitary–adrenal
CRH	Corticotrophin releasing factor
ACTH	Adrenocorticotrophic hormone
CNS	Central nervous system
CSF	Cerebrospinal fluid
GWAS	Genome-wide association studies
MHC	Major histocompatibility complex
CRP	C-reactive protein
IL	Interleukins
PANSS	Positive and Negative Syndrome Scale
TNF	Tumor necrosis factor
TGF	Transforming growth factor
IFN	Interferon
sIL-2R	Soluble IL-2-receptor
CDL5	Cluster of differentiation 5-like protein
CDL40	cluster of differentiation 40
NMDA	*N*-methyl-d-aspartate
NSAID	Nonsteroidal anti-inflammatory drugs
DST	Dexamethasone suppression test
CAR	Cortisol awakening response
SNPs	Single nucleotide polymorphisms
FKBP5	FK506 binding protein-5
CRHR1	Corticotrophin-releasing hormone receptor 1
GR	Glucocorticoid receptor
CRHBP	CRH binding protein
NC3R1	BclI polymorphism in glucocorticoid receptor gene 11
DHEA	dehydroepiandrosterone
DHEAS	DHEA sulphate
BMI	Body mass index
NGF	Nerve growth factor
BDNF	Brain-derived neurotrophic factor
NT-3	Neurotrophin-3
NT-4/5	Neurotrophin-4/5
p75NTR	Pan neurotrophin receptor
mRNA	messenger RNA
5-HTR2	Serotonergic receptor type 2
DRD2	Dopaminergic receptor type 2
NGFR	NGF receptor
DA	Dopamine
DR	Dopamine receptor i.e., DRD2-dopamine receptor type D2
PFC	Prefrontal cortex
TH	Tyrosine hydroxylase
DAT	Dopamine transporter
HVA	Homovanillic acid
COMT	Catechol-*O*-methyl transferase
LSD	Lysergic acid diethylamide
5-HT	Serotonin
5-HTT/SERT	Serotonin transporter
5-HTR	Serotonin receptor i.e., 5-HTR1A-serotonin receptor type 1A
MHPG	3-methoxy-4-hydroxyphenylglycol
NMDAR	*N*-methyl-d-aspartate receptor
mGluR	Metabotropic glutamate receptor i.e., mGluR3- Metabotropic glutamate receptor type 3
GABA	Gamma-amino-butyric acid
GAD	Glutamic acid decarboxylase
GABR	GABA-A receptor gene i.e., GABRB2-GABA-A receptor beta2 subunit gene
